# Compartmentalized Cytokine Responses in Hidradenitis Suppurativa

**DOI:** 10.1371/journal.pone.0130522

**Published:** 2015-06-19

**Authors:** Theodora Kanni, Vassiliki Tzanetakou, Athina Savva, Brigit Kersten, Aikaterini Pistiki, Frank L. van de Veerdonk, Mihai G. Netea, Jos W. van der Meer, Evangelos J. Giamarellos-Bourboulis

**Affiliations:** 1 4th Department of Internal Medicine, University of Athens, Medical School, Athens, Greece; 2 Department of Internal Medicine, Radboud University Medical Center and Nijmegen Institute for Infection, Inflammation and Immunity (N4i), Nijmegen, The Netherlands; Laikon Hospital, GREECE

## Abstract

**Background:**

Favorable treatment outcomes with TNF blockade led us to explore cytokine responses in hidradenitis suppurativa (HS).

**Methods:**

Blood monocytes of 120 patients and 24 healthy volunteers were subtyped by flow cytometry. Isolated blood mononuclear cells (PBMCs) were stimulated for cytokine production; this was repeated in 13 severe patients during treatment with etanercept. Cytokines in pus were measured.

**Results:**

CD14^bright^CD16^dim^ inflammatory monocytes and patrolling monocytes were increased in Hurley III patients. Cytokine production by stimulated PBMCs was low compared to controls but the cytokine gene copies did not differ, indicating post-translational inhibition. The low production of IL-17 was restored, when cells were incubated with adalimumab. In pus, high concentrations of pro-inflammatory cytokines were detected. Based on the patterns, six different cytokine profiles were discerned, which are potentially relevant for the choice of treatment. Clinical improvement with etanercept was predicted by increased production of IL-1β and IL-17 by PBMCs at week 8.

**Conclusions:**

Findings indicate compartmentalized cytokine expression in HS; high in pus but suppressed in PBMCs. This is modulated through blockade of TNF.

## Introduction

Hidradenitis suppurativa (HS) is a chronic devastating skin disorder affecting areas rich in apocrine glands. Nodules appear in the affected areas; they progressively become swollen and rupture with the release of pus. This process occurs repeatedly, ending to sinus tract formation and scars [1]. HS seems to indiscriminately affect the global population. Although the exact epidemiology is unknown, a recent large epidemiological survey in France reports 0.97% disease prevalence [2]. HS has considerable impact on the quality of life of patients, often leading to loss of many working hours per month. The Dermatology Quality Life Index (DQLI) for HS is 8.9, being thus higher than any other skin disorder [3].

We have previously demonstrated defective lipopolysaccharide (LPS)-induced production of the pro-inflammatory cytokines TNFα and IL-6 by blood monocytes of patients with HS [4]. This finding is a paradox since HS skin lesions are heavily inflamed and often they respond to anti-cytokine treatment either with agents blocking TNFα [5] or with agents blocking IL-1β and IL-1α [6]. Puzzled by this paradox, the current study was undertaken to better understand the role and the regulation of the various pro-inflammatory cytokines in HS. We wanted to know which monocyte populations and subpopulations (i.e. inflammatory monocytes and patrolling monocytes) are involved. Also we wanted to understand whether peripheral cytokine production is inhibited at the transcriptional or post-transcriptional level and whether this phenomenon of down-regulation is modulated by anti-TNF treatment. Finally, we aimed to find out the cytokine profile at the level of the inflammatory lesions, by measuring cytokines is pus.

## Methods

### Study population

The study was conducted during the period September 2009 to January 2012 in patients under follow-up in the Outpatient Department of Immunology and Infectious Diseases of the ATTIKON University Hospital (approval 103/24.03.2009). The study was approved by the Ethics Committee of the hospital. Written informed consent was provided by all patients and controls.

Diagnosis of HS was based on the following criteria: a) onset early after puberty; b) presence of subcutaneous nodules in areas of skin rich in apocrine glands; and c) a compatible history of recurrent drainage of pus from the affected areas [3, 7].

Clinical characteristics of patients were recorded comprising demographics, age since disease onset, involved areas and Hurley stage of severity. Lesions were graded according to the clinical system of Hurley [3, 7]. The severity of the disease was assessed during follow up of these patients according to the scoring system proposed by Sartorius et al [8]. Whole blood was collected from patients after venipuncture of one forearm vein under aseptic conditions. Blood sampling was repeated for some patients under treatment with anti-TNF agents. Blood sampling was also performed from 24 healthy volunteers. When available, pus was collected from the HS lesions, as indicated below.

### Flow cytometry

In order to study the monocyte subsets, four ml of whole blood were collected into ethylenediamine tetraacetic acid (EDTA)-coated tubes. Whole blood was incubated for 15 minutes in the dark with the flurochrome-conjugated monoclonal antibodies anti-CD14 (FITC, emission 525 nm, Immunotech, Marseille, France); anti-CD16 (PE, emission 525 nm, Immunotech); and anti-CD45 (PC5, emission 680 nm, Immunotech). Red blood cells were lysed (VersaLyse Solution, Immunotech, Marseille, France) and white blood cells were fixed with 0.16% formaldehyde (Fixative Solution, Immunotech). For each sample isotypic IgG controls were used. Cells were analyzed after running through the CYTOMICS FC flow cytometer (Beckman Coulter Co, Miami, Florida) with gating for monocytes based on their characteristic side scattering and CD45 expression. Absolute counts were determined using fluorospheres (Flow count, Immunotech).

### Cytokine production assays

Isolation of peripheral blood mononuclear cells (PBMCs) and stimulation for cytokine production were done as described elsewhere [9]. Cytokine stimuli were: 10 ng/ml of LPS of *Escherichia coli* O55:B5; 1x10^6^ colony forming units (cfu)/ml of heat-killed *Candida albicans* (HKCA); and 1x10^6^ cfu/ml of heat-killed *Staphylococcus aureus* (HKSA). HKCA and HKSA were prepared after heating 5x10^8^ cfu/ml of live isolates derived from pus cultures of lesions of patients with HS, for four hours at 70°C. Absence of growth was verified, after the end of heating, by subculture onto Saboureaud’s agar and onto blood agar respectively. The experiments were performed in the absence/presence of 10μg/ml of adalimumab (Abbott Laboratories, Chicago, IL). This concentration was selected because it is within the range of steady state serum concentrations after administration of conventional doses [10].

Concentrations of TNFα, IL-1β IL-6, IL-10 and IL-1ra were measured in supernatants of the 24-hour incubation; those of IL-17, IL-22 and human β-defensin-2 (hBD-2) in supernatants of the 5-day incubation. Cytokines were measured in duplicate by ELISA (R&D Minneapolis, USA). The lowest detection limits were: 40 pg/ml for TNFα; 20 pg/ml for IL-1β, IL-6, ΙL-10 and IL-1ra; 78 pg/ml for IL-17 and IL-22; and 6 pg/ml for hBD-2.

### Quantitative PCR for mRNA expression of IL-1β and TNFα

To measure cytokine specific mRNA, isolated PBMCs at a density of 2x10^6^/ml were left unstimulated or stimulated with1ng/ml LPS of *E*. *coli* O55:B5 for 4 hours at 37°C in 5% CO_2_ under the growth conditions described above into wells of 12-well plates_._ Then plates were centrifuged, supernatants were discarded and cells were lysed with Trizol (Invitrogen, Karlsruhe, Germany) and kept at −80°C until extraction of RNA. RNA extraction, synthesis of cDNA and measurements of gene transcripts was done as described elsewhere [9].

### Pus collection

Pus was collected from lesions of patients naïve to any therapy with TNF antagonists. The area of lesions was cleaned with sterile gauze moistened with normal saline 0.9% before the collection of pus. After prudent palpation of a representative draining fistula, a plastic 20G needle was put inside the fistula and 100 μl of pus were aspirated into an insulin syringe applied at the end of the needle. Pus was diluted with 900 μl of water for injection (dilution 1:10). The final volume of 1 ml was poured into a sterile Eppendorf tube and kept at -70°C until cytokine measurement. Concentrations of cytokines were measured as described above.

### Treatment with etanercept

Thirteen patients with Hurley III stage disease were given therapy with the TNF antagonist etanercept. None of the patients had been enrolled in the previous published trial of our group [11]. Ideally, these patients should have received treatment with adalimumab since adalimumab was added in the cultures of PBMCs. However, since results of a previous phase 2 clinical study of our group approved by the National Organization for Medicines of Greece showed considerable benefit from the use of etanercept in patients, the National Organization for Medicines of Greece approved only the off-label treatment with etanercept for these patients after application by the treating physician (author EGB). All patients provided written informed consent for this off-label treatment. Etanercept was administered subcutaneously at a dose of 50 mg twice weekly for 24 weeks. Before start of treatment all patients were thoroughly investigated so that they were bearing negative skin tuberculin test; negative chest X-ray; negative serology for human immunodeficiency virus (HIV), for hepatitis B virus (HBV) and for hepatitis C virus (HCV); negative case-history and family history for any demyelinating disorder; and normal liver biochemistry.

Blood sampling for isolation and stimulation of PBMCs and for separation of serum was done before start of treatment and eight weeks from start of treatment. This time point was arbitrarily chosen. At the end of treatment patients were divided into responders if they had a decrease of Sartorius score by more than 30% from the baseline, or non-responders when patients experienced persistence of pain and purulence from the affected skin areas. Concentrations of C-reactive protein (CRP) were measured in serum samples by an immunoturbidometric assay (Roche, Paris, France, lower detection limit 3.2 mg/l).

### Statistical analysis

Results were expressed as means ± SE. Comparisons of the absolute counts of monocyte subsets between healthy controls and patients at different Hurley stages were done by ANOVA with *post-hoc* analysis by Bonferroni. Comparisons of cytokine stimulation between healthy controls and patients were done by the Mann-Whitney U test. Comparisons of treated PBMCs in the absence and in the presence of adalimumab were performed using the Wilcoxon’s rank signed test. Percent changes of cytokine production from the baseline on the eighth week from start of treatment with TNF antagonists were measured. Comparisons between patients who did not improve and patients who improved were done by the Mann Whitney U test. Statistical correlations were done according to Spearman’s rank of order. Median concentration of each cytokine was determined in pus. Based on this measurement patients were divided with high production (above the median) and low production (below the median) of each specific cytokine. After cross-tabulation and TURF analysis, patients were classified according to the expressed pattern of cytokines. Any value of p below 0.05 after adjustment for multiple comparisons by Bonferroni was considered significant (S1 File).

## Results

### Study population

A total of 120 patients with HS were enrolled in the study; 39 patients were classified as stage I disease according to Hurley; 37 patients as stage II disease; and 44 patients as stage III disease. Their characteristics are shown in Table 1. Mean ± SD age of controls was 37.3 ± 11.1 years (p: 0.163 compared with patients); nine controls were male and fifteen were female (p: 0.232 compared with patients).

**Table 1:** Clinical characteristics of patients enrolled in the study.

Number of patients	120
Age (years, mean ± SD)	37.3 ± 5.9
Male/Female (number, %)	43 (35.8)/77(64.2)
Years from initial diagnosis (median, range)	10.0 (1.0–35.0)
Disease stage (number, %)	
Hurley I	39 (32.5)
Hurley II	37 (30.8)
Hurley III	44 (36.7)
Sartorius score by Hurley stage (mean ± SD)	
Hurley I	28.1 ± 20.2
Hurley II	52.4 ± 24.9
Hurley III	129.3 ± 79.2
Involved body areas (number, %)	
Axillae	66 (55.0)
Submammary folds	30 (25.0)
Femoral folds	96 (80.0)
Groins	47 (39.2)
Perianal area	53 (44.2)

### Monocyte subpopulations

**Fig 1:**
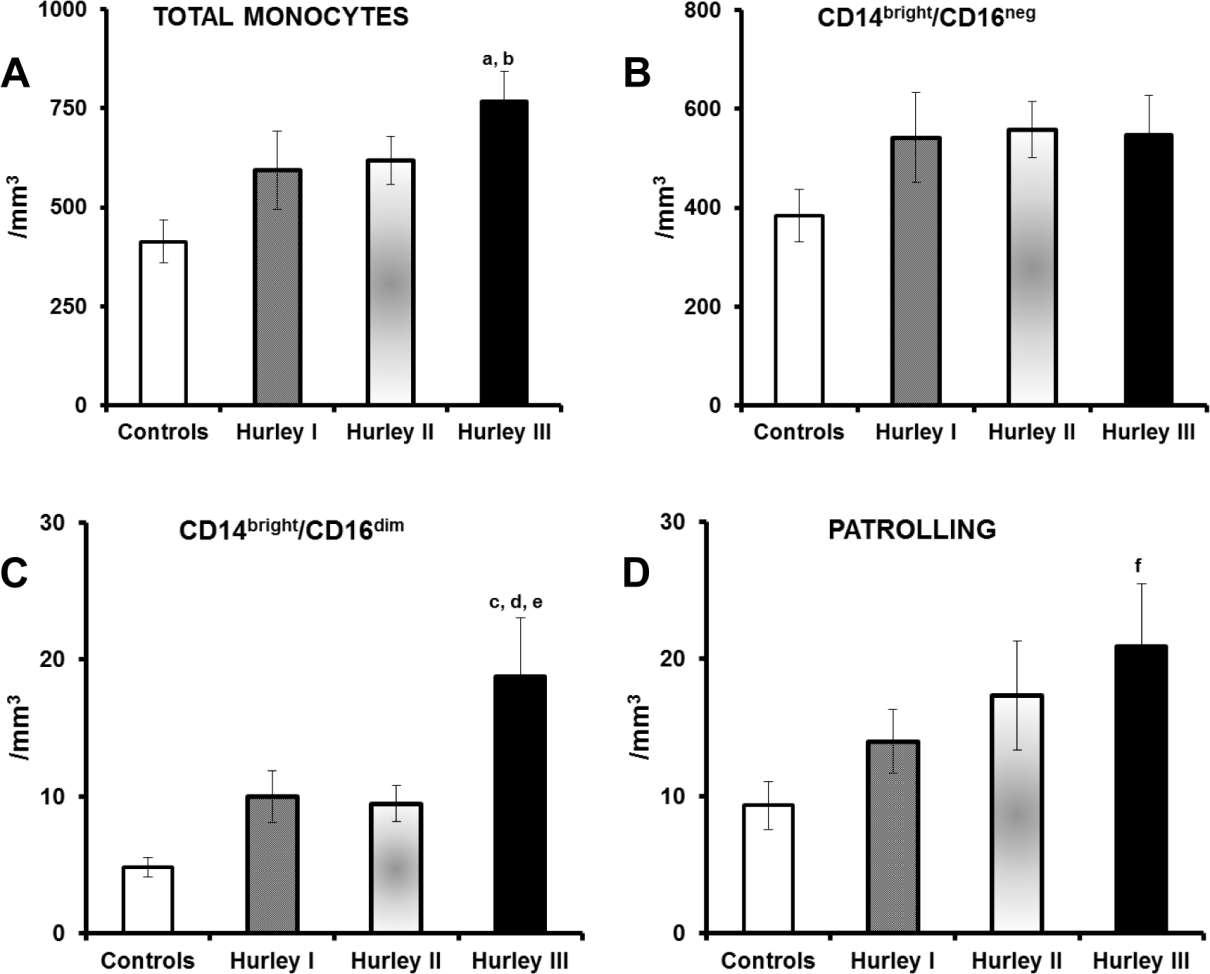
Monocyte subsets in HS. Absolute counts of (A) total circulating monocytes, (B) CD14^bright^/CD16^neg^ monocytes, (C) CD14^bright^/CD16^dim^ monocytes, and (D) patrolling monocytes are shown for healthy volunteers (n = 14), patients with Hurley I HS (n = 37), with Hurley II HS (n = 37) and with Hurley III HS (n = 44). P values of comparisons by ANOVA and *post hoc* Bonferroni corrections: ^a^0.004 vs healthy controls; ^b^0.026 vs Hurley I; ^c^0.001 vs healthy controls; ^d^0.012 vs Hurley I; ^e^0.008 vs Hurley II; ^f^0.037 vs healthy controls.

The absolute count of subpopulations of circulating monocytes in patients and in healthy volunteers is given in Fig 1. There was a tendency of an increase in total numbers of monocytes, as well as in monocytes with the inflammatory phenotype CD14^bright^CD16^dim^ and in patrolling monocytes (CD14^negative^CD16^bright^ or CD14^dim^CD16^bright^) among HS patients. The increases were significant for the Hurley stage III patients. A positive correlation was found between the absolute count of monocytes and the Sartorius score (r_s_: +0.361, p: 0.003). Since inflammatory monocytes are considered as the main producers of pro-inflammatory cytokines, it was concluded that the low cytokine production by these cells described previously [4] was not due to a depletion of these cells.

### Cytokine production

**Fig 2:**
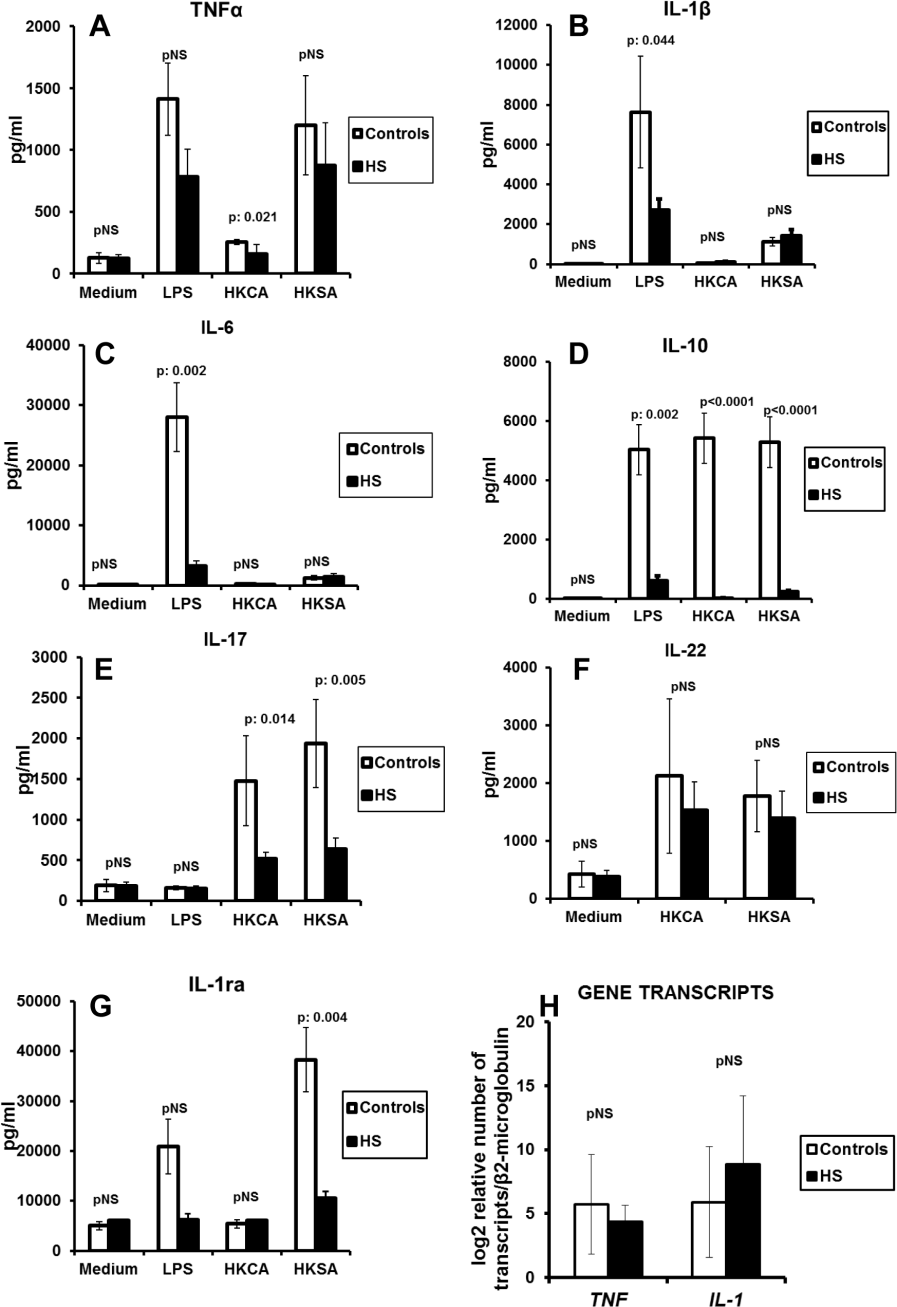
Stimulation of circulating monocytes. Production of (A) tumour necrosis factor-alpha (TNFα), (B) interleukin (IL)-1β, (C) IL-6, (D) IL-10, (E) IL-10, (F) IL-22, and (G) IL-1ra by peripheral blood mononuclear cells (PBMCs) of eight healthy controls and of 80 patients with hidradenitis suppurativa (HS). PBMCs were stimulated with LPS of *Escherichia coli* O55:B5; with heat-killed *Candida albicans* (HKCA), and with heat-killed *Staphylococcus aureus* (HKSA). (H) Gene transcripts of *TNF* and of *IL-1* after stimulation with LPS. P values refer to comparisons with healthy controls by the Mann-Whitney U test; pNS: difference not-significant.

The production of TNFα, IL-1β, IL-6, IL-10, IL-17, IL-22 and IL-1ra by PBMCs of healthy controls and by PBMCs of patients with HS is shown in Fig 2. Production of cytokines by PBMCs of patients was low compared to controls. This was not only the case for the pro-inflammatory cytokines but also for the inhibitory cytokines IL-1ra and IL-10. This phenomenon appeared to be dependent to some extent of the applied stimulus. Production of TNFα was decreased after stimulation with HKCA (Fig 2A); that of IL-1β and IL-6 after stimulation with LPS (Fig 2B and 2C); that of IL-1ra and of IL-10 with all three studied stimuli (Fig 2D and 2G); and that of IL-17 after stimulation with HKCA and HKSA (Fig 2E). Production of hBD-2 was below the lower limit of detection in all samples (data not shown).

As shown in Fig 2H, transcripts of *TNF* and of *IL-1β* did not differ between HS patients and healthy volunteers, pointing out that there is an inhibition at the post-transcriptional level.

**Fig 3:**
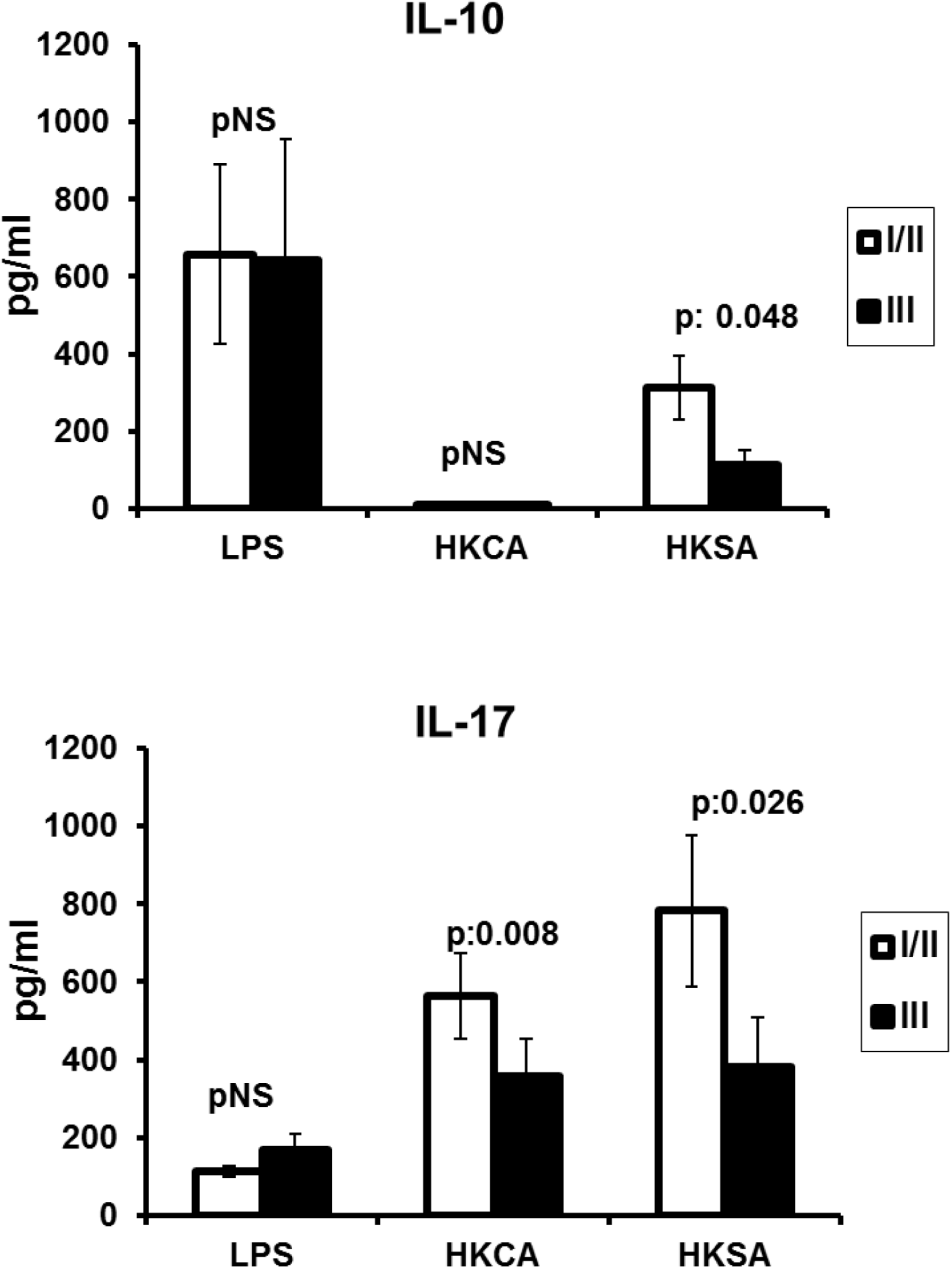
Stimulation of IL-10 and IL-17 in relation with disease severity Production of IL-10 and IL-17 was stimulated in peripheral blood mononuclear cells (PBMCs) of patients with hidradenitis suppurativa (HS) by LPS of *Escherichia coli* O55:B5, heat-killed *Candida albicans* (HKCA) and heat-killed *Staphylococcus aureus* (HKSA). Patients are divided into those with Hurley I or Hurley II disease stage (n = 50) and into those with Hurley III disease stage (n = 40). P values refer to comparisons between disease stages by the Mann Whitney U test; pNS: difference not-significant.

For each of the defective cytokine responses to the stimuli mentioned, we assessed whether they are related to the Hurley disease stage. We compared patients in Hurley stages I/II with the most severe stage Hurley III. Patients at Hurley III disease stage had impaired production for IL-17 and to a lesser extent for IL-10 (Fig 3). Significant differences were not found for the other cytokines between patients with Hurley I/II and patients with Hurley III stage (data not shown).

**Fig 4:**
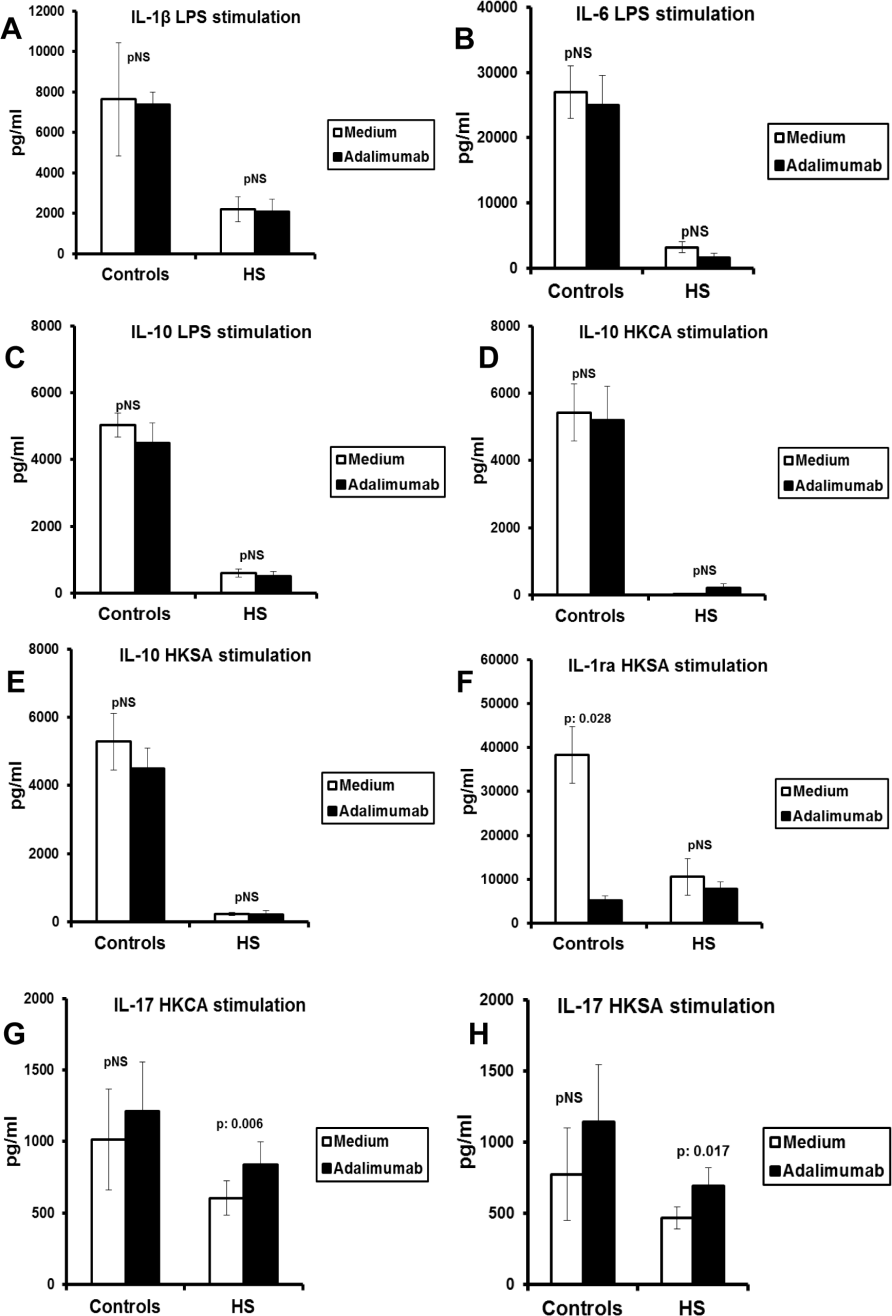
Modulation of cytokine release by adalimumab. Peripheral blood mononuclear cells (PBMCs) from of eight healthy controls and 40 patients with hidradenitis suppurativa were stimulated for the production of (A) interleukin (IL)-1β with LPS of *Escherichia coli* O55:B5; (B) IL-6 with LPS of *E*.*coli* O55:B5; (C) IL-10 with LPS of *E*.*coli* O55:B5; (D) IL-10 with heat-killed *Candida albicans* (HKCA); (E) IL-10 with heat-killed *Staphylococcus aureus* (HKSA); (F) IL-1ra with HKSA; (G) IL-17 with HKCA; (H) IL-17 with HKSA. P values refer to comparisons of cytokine production without/with adalimumab; pNS: difference not-significant.

Thereafter, we investigated whether defective cytokine production is changed when the cells are co-incubated with the anti-TNF agent adalimumab (Fig 4). The production of IL-17 after stimulation with either HKCA or HKSA significantly increased in the presence of adalimumab. Such changes were not seen for any other cytokine.

### Cytokine concentrations in pus

**Fig 5:**
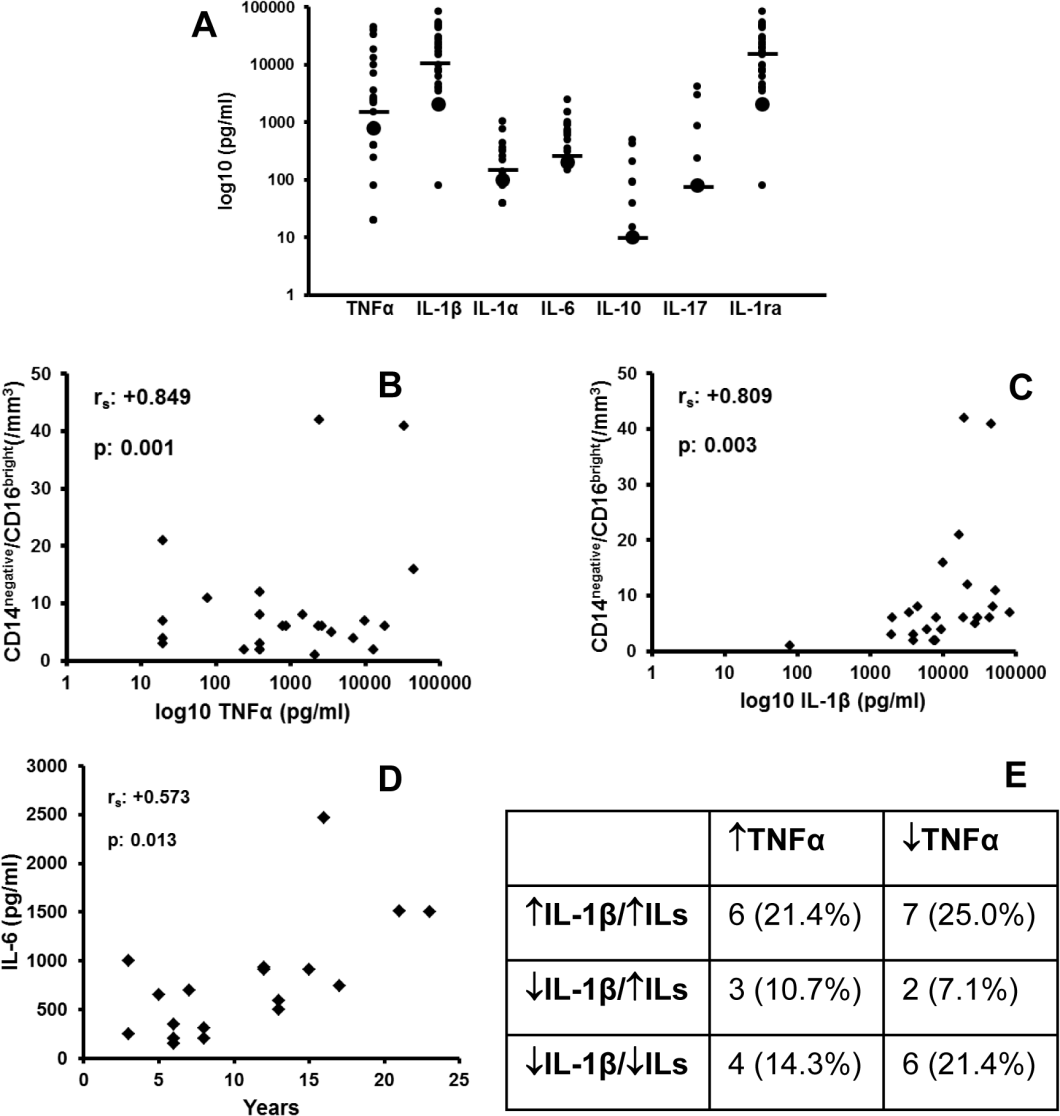
Cytokine concentrations in pus of patients with HS. (A) Concentrations of tumour necrosis factor-alpha (TNFα), interleukin (IL)-1β, IL-1α, IL-6, IL-10, IL-17 and Il-1ra were measured in the pus of the lesions of 28 patients of Hurley III disease stage. Lines represent medians. Correlations between (Β) the number of patrolling monocytes and pus TNFα; (C) the number of patrolling monocytes and pus IL-1β; and (D) IL-6 and years since disease onset. Classification patterns of patients according to pus cytokines is shown in panel E. Spearman’s coefficients of correlation and p values of significance are provided.

The next step was to obtain pus from patients with HS and measure cytokines in these samples. As can be seen in Fig 5A, sizable concentrations of TNFα, IL-1β and IL-1ra were present in the pus of all patients and none of the patients had concentrations of any of these cytokines below the lower detection limit. However, 25.0% of patients had pus IL-1α below the detection limit, 15.4% had pus IL-6 below the detection limit, 71.4% had pus IL-10 below the lower detection limit and 78.6% had pus IL-17 below the detection limit. Furthermore, positive correlations were found between the absolute counts of circulating patrolling monocytes and the concentrations of TNFα (r_s_: +0.675, p: 0.032) and of IL-1β (r_s_: +0.778, p: 0.008) in pus (Fig 5B and 5C). The years since onset of HS correlated positively with IL-6 in pus and not with the other cytokines (Fig 5D).

According to the concentrations of cytokines in pus, patients were classified into six patterns of cytokine expression taking into consideration if patients had high or low concentrations of TNFα, high or low concentrations of IL-1β and high or low concentrations of any one of the other five measured interleukins (Fig 5E). The most common expression patterns were i) low TNFα, high IL-1β and high ILs found in 25% of patients; ii) high TNFα, high IL-1β and high ILs found in 21.4% of patients; and iii) low TNFα, low IL-1β and low ILs found in21.4% of patients.

### Effect of treatment with etanercept on cytokine production

**Fig 6:**
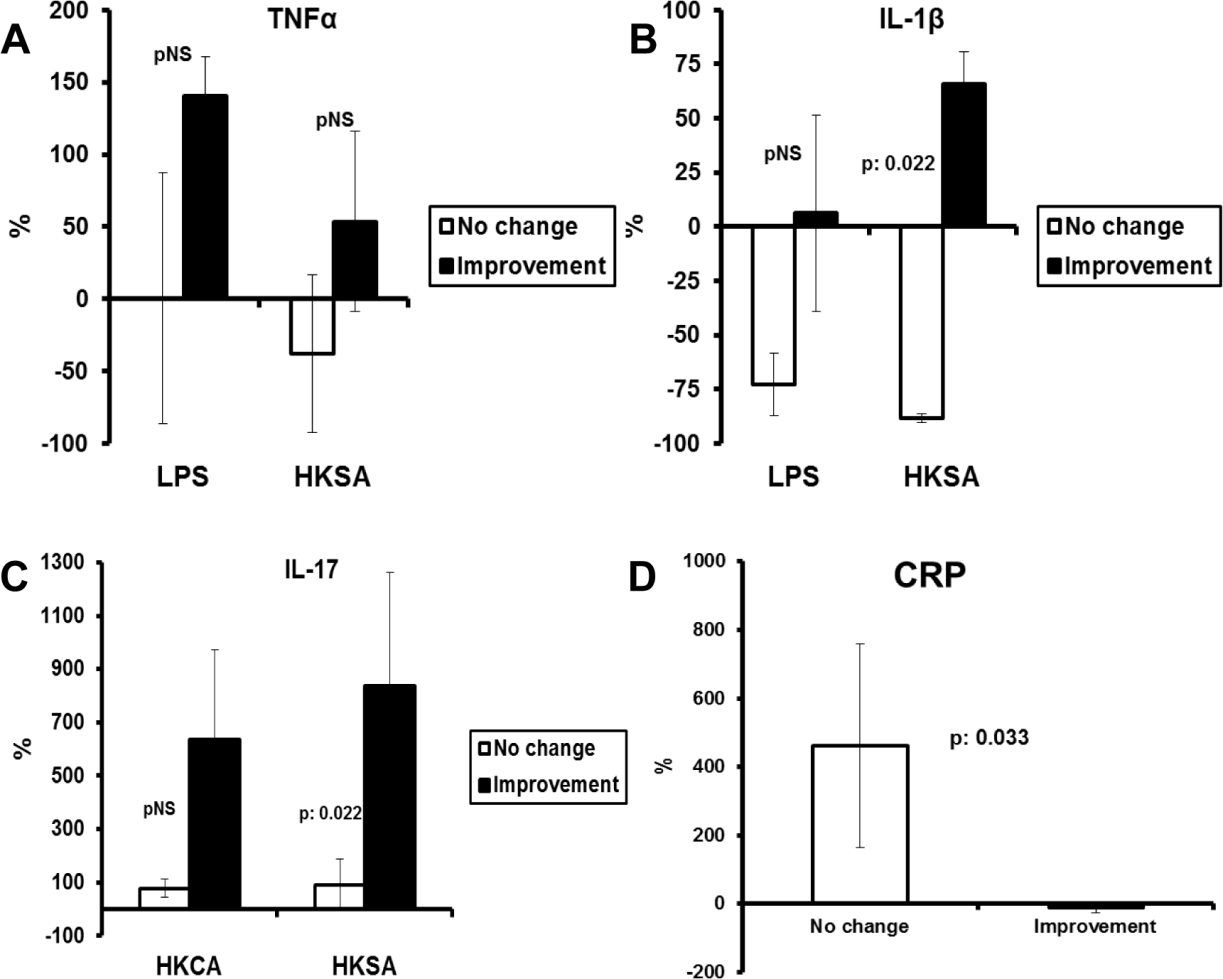
Changes of cytokine production and of C Reactive Protein with the clinical course of HS. PBMCs were isolated and serum was sampled from patients with Hurley III disease at baseline and after 8 weeks of treatment with etanercept. Patients are divided into those experiencing worsening of symptoms (n = 6) or clinical improvement (n = 7) at the end of treatment. No change was defined when disease flare-ups persisted with heavy purulence. Improvement was defined as decrease of Sartorius score more than 30% from the baseline. PBMCs were stimulated with LPS of *Escherichia coli* O55:B5; with heat-killed *Candida albicans* (HKCA); and with heat-*Staphylococcus aureus* (HKSA) for the production of (A) tumour necrosis factor-alpha (TNFα), (B) interleukin (IL)-1β, (C) IL-17 and (D) C Reactive Protein (CRP). P values refer to comparisons of change of cytokine production from the baseline between improved and worsened cases by the Mann-Whitney U test.

To investigate the effect of anti-TNF treatment, PBMCs of 13 Hurley stage III patients were investigated for cytokine production before and during treatment. Production of cytokines tended to increase after treatment if the patient improved; lack of improvement was associated with a tendency to decreased production (Fig 6A, 6B and 6C). For the HKSA-induced production of IL-1β and IL-17 these changes were significant. Serum concentrations of CRP were increased in patients without improvement (Fig 6D).

## Discussion

In the present study, we have found that pus derived from lesions of patients with HS contains high concentrations of pro-inflammatory cytokines (TNFα, IL-1β, IL-1α, IL-17) and of anti-inflammatory cytokines (IL-10 and IL-1ra). Surprisingly, the profile of these cytokines differs considerably between patients. Monocytes from the circulation of HS patients are predominantly of the inflammatory phenotype, but they are refractory to respond to the conventional stimuli to produce cytokines. The refractoriness is regulated at the post-transcriptional level, since there are high specific mRNA concentrations present in these monocytes.

Our finding that different patients exhibit different cytokine profiles in pus is important for several reasons. First of all, it teaches us that the defect is not residing in the regulation of just one of these cytokines. This is unlike the situation in most auto-inflammatory disorders, such as familial Mediterranean fever, the cryopyrin-associated periodic syndromes and PAPA (pyogenic arthritis, pyoderma gangrenosum and acne), which are all IL-1β-mediated diseases [12]. Secondly, this is the first study to directly assess the presence of cytokines in HS pus. Our findings of high cytokine concentrations at the inflammatory site are in agreement with those of van der Zee et al [13] who cultured biopsies of lesional skin in HS and measured their ex-vivo cytokine production. They found increased immune reactivity for TNFα, IL-β and IL-10 in cultures of the dermis and epidermis of 20 patients. However, they did not try to classify patients according to the cytokine profile in the lesions. Thirdly, the finding that patients show different profiles of cytokines in pus does not come as a complete surprise: Endres et al, many years ago, could demonstrate that healthy volunteers differ with regard to the most prominent cytokine response of their PBMCs in vitro, pointing to personalized cytokine profiles [14].

An important question is whether we may be able to predict the response to treatment with cytokine inhibitors based on the profile in the pus. In other words, would a patient who does not exhibit a high concentration of TNFα in the pus fail to respond to anti-TNF treatment? It is not possible to make such a statement yet, given the relatively low number of observations. Reported clinical efficacy of treatment with TNF antagonists is variable between studies which have enrolled small number of patients [4, 15–20]. In the first large trial of weekly treatment with the monoclonal antibody adalimumab, considerable efficacy was shown. Although the primary study endpoint based on changes of the physicians’ global assessment score showed 17.9% treatment benefit compared to 3.9% of the placebo group [5], patients treated with adalimumab experienced considerable improvement in all secondary study outcomes i.e. reduction of the total number of inflammatory nodules and decreases of the DLQI and of the modified Sartorius scores. This implies that not all patients respond to anti TNF treatment and other modes of treatment have to be explored. To that end we recently completed a pilot randomized controlled study on anakinra in patients with HS [21]

In all cases, it would be a great benefit if clinicians would be able to predict which patients are likely to respond to which of these expensive treatment options. From the presented data only the reversal of the suppression of production of IL-1β and IL-17 by circulating PBMCs after stimulation with HKSA may be helpful to this end. Reversal of the inhibited production of these two cytokines is found within the first eight weeks after start of treatment with etanercept and it is a predictor of favorable clinical responses at the end of the 24-week period of treatment. Our findings cannot be generalized since etanercept has not documented clinical efficacy in large-scale trials. However, they are promising for the development of some bioassay to predict favorable clinical responses in HS.

Our findings regarding the suppressed cytokine production in PBMCs from HS patients concur with our previous findings of a decreased LPS-induced production of TNFα and of IL-6 ex vivo in HS patients [4]. Here we extend these findings, showing that it concerns a down-regulation that is not limited to LPS, but also occurs when the cells are exposed to heat-killed *S*. *aureus* and *C*.*albicans* that bear ligands interacting with receptors other than Toll-like receptor 4. We also demonstrate that production of IL-17, a cytokine mainly produced by T helper 17 lymphocytes and chemo-attracting neutrophils [22], is down-regulated. This means that the down-regulation of the cytokine responses is not limited to monocyte-derived cytokines. The IL-17 production was inversely correlated with the severity of the disease.

The findings of down-regulated ex-vivo cytokine production in an inflammatory disease like HS are reminiscent of the state of tolerance observed during acute severe infections [23] and with exposure to bacterial endotoxin (LPS) [24]. In these conditions the circulating cytokines may be found elevated in the early phase of the disease, to be followed by a preponderant anti-inflammatory state. In a recent study by Matusiak et al [25], elevated serum concentrations of TNFα have been reported in patients with HS. These high circulating levels of TNFα may be responsible, at least in part, for the down-regulation of cytokine production by PBMCs after ex vivo stimulation.

**Fig 7:**
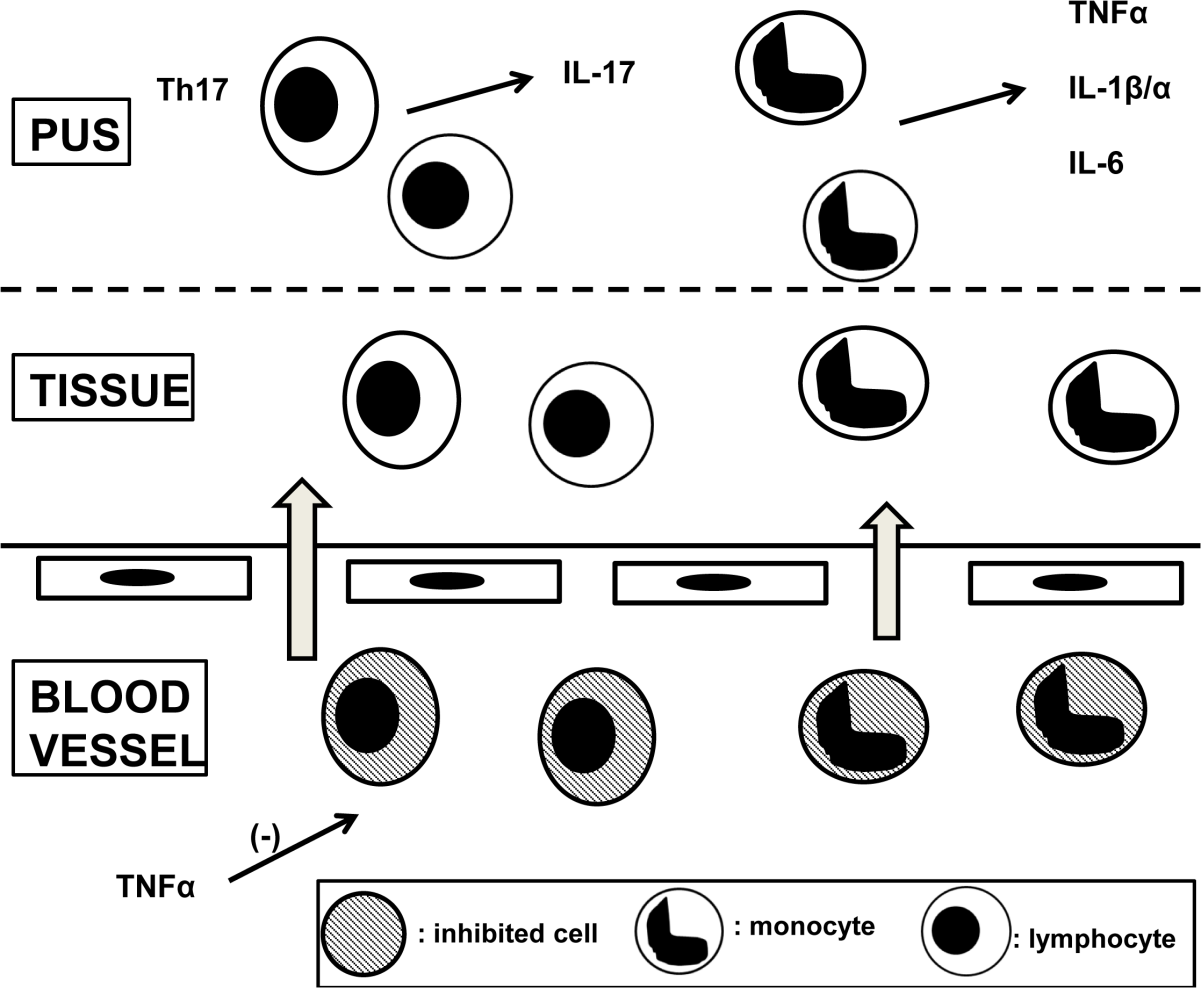
A proposed scheme of pathogenesis of HS. Patients with HS have monocytosis but cytokine responses of inflammatory monocytes and T17 lymphocytes are inhibited. This inhibition is mediated for T17 cells by tumour necrosis factor-alpha (TNFα). When cells migrate to tissues from the blood vessels through the vascular endothelium, capacity for excess production of TNFα, interleukin (IL)-1β, IL-1α, IL-6 and IL-17 is restored.

Several decades of research have greatly enhanced our understanding of the molecular mechanisms of endotoxin tolerance [24]. However it is currently unclear how these relate to the state of tolerance described here; the exact mechanisms cannot be easily elucidated in clinical material, but nevertheless further research is necessary. The tolerance in HS pertains to the blood and is a feature of a compartmentalized cytokine response, in which the bloodstream is protected against cytokine action, and monocytes recruited to the site of inflammation appear to lose their tolerant state (Fig 7). If this hypothesis is correct, then improvement of skin inflammation with anti-TNF treatment will reverse the inhibition exerted on circulating monocytes towards increased cytokine production. This is in agreement with the reversal of the inhibition of the production of IL-1β and IL-17 by PBMCs seen in patients experiencing clinical benefit from anti-TNF treatment. Our data also demonstrate that tolerance is not a consequence of a depletion of inflammatory monocytes from the bloodstream. In fact, a tendency towards monocytosis was detected. It was also shown that when the monoclonal antibody against TNFα adalimumab was added to the in-vitro cultures, it partially restored the decreased IL-17 production. This suggests that the tolerance, at least for IL-17 production is driven in part by TNFα either as a soluble ligand or in its membrane-bound form.

Our study presents two major limitations. The first is the lack of measurement of cytokines in the skin of healthy volunteers. This was not feasible because cytokine were measured in the pus of lesions and healthy volunteers do not have visible skin breaches to allow sampling. The second is the observational nature of the study so that findings should be interpreted with caution.

## Conclusions

The present study shows that in HS production of pro-inflammatory cytokine is compartmentalized; high in skin but decreased from circulating PBMCs. Reversal of the inhibited production of IL-1β and IL-17 by circulating PBMCs within the first 8 weeks of therapy may be one predictor of favorable response to TNF antagonists.

## Supporting Information

S1 File Study dataset. (PDF)
