# Supplementary material for: Compartmentalized Cytokine Responses in Hidradenitis Suppurativa
Source: PLoS One. 2015 Jun 19;10(6):e0130522. doi: 10.1371/journal.pone.0130522 (PMC4474720; doi:10.1371/journal.pone.0130522)
Supplement: S1 File — (PDF) [file pone.0130522.s001.pdf]

group	stage	gender	age	il1medium	il1lps1	il1candida	il1staph	il1mediuma
Controls	Controls	Male	25,00	75,00	1560,00	70,00	378,00	20,00
Controls	Controls	Female	26,00	65,00	7040,00	60,00	1700,00	40,00
Controls	Controls	Male	34,00	45,00	5000,00	60,00	1880,00	30,00
Controls	Controls	Male	42,00	35,00	4780,00	85,00	713,00	20,00
Controls	Controls	Male	36,00	20,00	603,00	35,00	775,00	20,00
Controls	Controls	Male	37,00	193,00	2080,00	45,00	605,00	100,00
Controls	Controls	Male	27,00	78,00	20000,00	70,00	1145,00	40,00
Controls	Controls	Male	27,00	40,00	20000,00	83,00	1780,00	25,00
Controls	Controls	Male	32,00	20,00	1840,00		875,00	
Controls	Controls	Male	32,00	20,00	5000,00		810,00	
Controls	Controls	Male	42,00	20,00	2415,00		765,00	
Controls	Controls	Male	42,00	240,00	400,00		20,00	
Controls	Controls	Female	27,00	40,00	2030,00		640,00	
Controls	Controls	Female	27,00	20,00	4520,00		1630,00	
Controls	Controls	Female	34,00	20,00	1030,00		1610,00	
Controls	Controls	Female	35,00					
Controls	Controls	Female	31,00					
Controls	Controls	Female	45,00					
Patients	Hurley II	Female	53,00					
Patients	Hurley I	Female	20,00					
Patients	Hurley I	Female	17,00					
Patients	Hurley II	Female	59,00					
Patients	Hurley I	Female	33,00					
Patients	Hurley III	Male	23,00					
Patients	Hurley III	Female	38,00					
Patients	Hurley II	Female	38,00					
Patients	Hurley II	Female	47,00	230,00	5420,00			
Patients	Hurley III	Male	36,00	20,00	208,00	20,00	148,00	20,00
Patients	Hurley III	Male	57,00	50,00	10000,00		450,00	
Patients	Hurley II	Female	34,00					
Patients	Hurley III	Female	48,00					
Patients	Hurley II	Male	45,00					
Patients	Hurley III	Female	47,00					
Patients	Hurley II	Male	69,00					
Patients	Hurley I	Female	45,00					
Patients	Hurley III	Male	28,00					
Patients	Hurley III	Male	46,00					
Patients	Hurley III	Male	25,00					
Patients	Hurley II	Male	27,00					
Patients	Hurley I	Female	29,00					
Patients	Hurley I	Male	36,00					
Patients	Hurley I	Female	26,00					
Patients	Hurley II	Female	25,00					
Patients	Hurley II	Female	48,00					
Patients	Hurley II	Female	36,00					
Patients	Hurley II	Female	50,00					

Patients	Hurley II	Female	44,00					
Patients	Hurley III	Female	35,00					
Patients	Hurley II	Male	44,00					
Patients	Hurley II	Female	12,00					
Patients	Hurley II	Female	17,00					
Patients	Hurley III	Male	50,00					
Patients	Hurley II	Female	17,00					
Patients	Hurley III	Male	32,00	265,00	620,00			
Patients	Hurley I	Female	36,00					
Patients	Hurley I	Male	23,00					
Patients	Hurley I	Male	31,00					
Patients	Hurley III	Male	52,00					
Patients	Hurley I	Female	48,00					
Patients	Hurley II	Male	41,00					
Patients	Hurley III	Female	55,00	240,00	5000,00		1665,00	
Patients	Hurley I	Female	62,00					
Patients	Hurley III	Female	37,00	225,00	703,00		390,00	
Patients	Hurley II	Male	24,00					
Patients	Hurley III	Male	21,00					
Patients	Hurley II	Female	30,00					
Patients	Hurley II	Female	54,00					
Patients	Hurley I	Male	44,00					
Patients	Hurley III	Male	47,00					
Patients	Hurley I	Female	43,00					
Patients	Hurley III	Female	50,00					
Patients	Hurley I	Female	20,00					
Patients	Hurley III	Male	53,00	100,00	5000,00	130,00	3000,00	93,00
Patients	Hurley III	Male	27,00	1128,00	1360,00	195,00	4400,00	30,00
Patients	Hurley I	Female	51,00	1128,00	1770,00	155,00	2738,00	30,00
Patients	Hurley I	Female	30,00					
Patients	Hurley I	Female	50,00					
Patients	Hurley II	Male	28,00					
Patients	Hurley II	Female	44,00					
Patients	Hurley II	Female	37,00					
Patients	Hurley III	Male	50,00					
Patients	Hurley III	Female	57,00					
Patients	Hurley II	Female	59,00					
Patients	Hurley III	Male	29,00					
Patients	Hurley II	Female	32,00					
Patients	Hurley I	Male	30,00					
Patients	Hurley II	Female	28,00					
Patients	Hurley II	Female	39,00					
Patients	Hurley II	Female	41,00					
Patients	Hurley II	Male	32,00					
Patients	Hurley II	Female	50,00					
Patients	Hurley II	Male	35,00					
Patients	Hurley II	Female	36,00					

Patients	Hurley III	Female	56,00	310,00	198,00			
Patients	Hurley III	Male	68,00					
Patients	Hurley III	Male	48,00					
Patients	Hurley I	Female	30,00					
Patients	Hurley II	Male	35,00	20,00		20,00	2060,00	
Patients	Hurley III	Male	36,00	220,00	2215,00			
Patients	Hurley II	Male	37,00					
Patients	Hurley II	Female	54,00					
Patients	Hurley II	Female	31,00					
Patients	Hurley III	Male	31,00					
Patients	Hurley III	Female	43,00	20,00	1700,00	90,00	535,00	20,00
Patients	Hurley II	Male	54,00					
Patients	Hurley III	Female	24,00					
Patients	Hurley III	Male	30,00	258,00	12400,00	260,00	1900,00	345,00
Patients	Hurley II	Male	21,00					
Patients	Hurley II	Male						
Patients	Hurley II	Female	37,00					
Patients	Hurley III	Male	51,00	195,00	1198,00		1458,00	
Patients	Hurley II							
Patients	Hurley III	Male	20,00	1185,00	520,00	455,00	983,00	480,00
Patients	Hurley III	Female	38,00					
Patients								
Patients								
Patients	Hurley I	Female	23,00					
Patients	Hurley I	Female	30,00					
Patients	Hurley I	Female	30,00					
Patients	Hurley I	Female	27,00					
Patients	Hurley I	Female	40,00					
Patients	Hurley II	Female	22,00					
Patients	Hurley III	Male	42,00	1620,00	4120,00	428,00	3920,00	75,00
Patients	Hurley I	Female	29,00					
Patients	Hurley III	Female	48,00					
Patients	Hurley I	Male	32,00					
Patients	Hurley I	Male	28,00					
Patients	Hurley III	Female	46,00					
Patients	Hurley III	Male	38,00					
Patients	Hurley III	Female	44,00					
Patients	Hurley III	Female	21,00					
Patients	Hurley I	Female	30,00					
Patients	Hurley II	Female	48,00					
Patients	Hurley I	Female	23,00					
Patients	Hurley III	Female	35,00					
Patients	Hurley I	Female	44,00					
Patients	Hurley I	Female	50,00					
Patients	Hurley I	Female						
Patients	Hurley I	Female	39,00	215,00	7080,00			
Patients	Hurley III	Female	63,00	20,00	265,00	20,00	168,00	20,00

Patients	Hurley III	Female	27,00					
Patients	Hurley III	Male	52,00					
Patients	Hurley II	Male	31,00					
Patients	Hurley II	Female	36,00					
Patients	Hurley II	Female	55,00					
Patients	Hurley III	Male	26,00					
Patients	Hurley II	Male	27,00					
Patients	Hurley III	Male	24,00	20,00	95,00	20,00	255,00	20,00
Patients	Hurley III	Female	41,00					
Patients	Hurley III	Female	37,00	278,00	6180,00			
Patients	Hurley III	Female	42,00					
Patients	Hurley III	Female		20,00	1725,00		850,00	
Patients	Hurley III	Male	25,00					
Patients	Hurley III	Male	59,00					
Patients	Hurley II	Female	33,00					
Patients	Hurley II	Female	35,00					
Patients	Hurley III	Female	23,00	20,00	338,00	45,00	343,00	20,00
Patients	Hurley III	Female	28,00					
Patients	Hurley II	Female	51,00					
Patients	Hurley II	Female	36,00	20,00	1283,00	20,00	590,00	20,00
Patients	Hurley II	Female	27,00					
Patients	Hurley II	Male	34,00					
Patients	Hurley II	Female	46,00	20,00	20,00	20,00	20,00	20,00
Patients	Hurley II	Female	54,00					
Patients	Hurley II	Male	37,00	40,00	1285,00	70,00	2200,00	35,00
Patients	Hurley I	Female	32,00					
Patients	Hurley II	Female	24,00					
Patients	Hurley II	Female	35,00					
Patients	Hurley I	Female	65,00					
Patients	Hurley II	Female	42,00					
Patients	Hurley II	Female	20,00					
Patients	Hurley II	Male	64,00					
Patients	Hurley II	Female	38,00					
Patients	Hurley II	Female	44,00					
Patients	Hurley I	Female	32,00					
Patients	Hurley II	Female	37,00					
Patients	Hurley I	Female	27,00					
Patients	Hurley I	Female	46,00					
Patients	Hurley III	Female	63,00					
Patients	Hurley II	Female	51,00					
Patients	Hurley III	Female	32,00					
Patients	Hurley III	Male	30,00					
Patients	Hurley II	Female	35,00	20,00	2270,00		640,00	
Patients	Hurley II	Female	37,00					
Patients	Hurley II	Female	41,00	20,00	485,00		1590,00	
Patients	Hurley III	Female	33,00					
Patients	Hurley I	Female	48,00					

Patients	Hurley II	Male	27,00					
Patients	Hurley I	Female	61,00					
Patients	Hurley II	Female	32,00					
Patients	Hurley I	Female	26,00					
Patients	Hurley I	Male	45,00					
Patients	Hurley I	Female	27,00					
Patients	Hurley II	Male	49,00	80,00	20,00		20,00	
Patients	Hurley III	Female	44,00	205,00	4420,00		5860,00	
Patients	Hurley III	Male	32,00					
Patients	Hurley III	Female	27,00					
Patients	Hurley I	Male	32,00					
Patients	Hurley III	Female	23,00					
Patients	Hurley II	Female	28,00					
Patients	Hurley I	Female	55,00	20,00	20,00	20,00	135,00	20,00
Patients	Hurley II	Female	40,00					
Patients	Hurley III	Female	56,00					
Patients	Hurley I	Female	18,00	20,00	1980,00	20,00	153,00	20,00
Patients	Hurley III	Female	37,00					
Patients	Hurley I	Female	45,00					
Patients	Hurley I	Male	23,00	30,00	2430,00		725,00	
Patients	Hurley I	Female	39,00					
Patients	Hurley I	Female	28,00					
Patients	Hurley I	Male	46,00					
Patients	Hurley II	Male	49,00					
Patients	Hurley II	Female	27,00					
Patients	Hurley II	Female	46,00					
Patients	Hurley II	Female	37,00	203,00	4320,00	303,00	1193,00	45,00
Patients	Hurley III	Female	64,00	40,00	1018,00	75,00	483,00	50,00
Patients	Hurley II	Male	31,00					
Patients	Hurley II	Male	42,00					
Patients	Hurley II	Male	33,00					
Patients	Hurley I	Female	20,00					
Patients	Hurley III	Male	53,00					
Patients	Hurley I	Male	40,00					
Patients	Hurley I	Male	42,00					
Patients	Hurley III	Female	42,00	20,00	2180,00	115,00	1075,00	20,00
Patients	Hurley II	Male	37,00					
Patients	Hurley III	Male	70,00					
Patients	Hurley II	Female	49,00					
Patients	Hurley II	Female	26,00					
Patients	Hurley III	Male	60,00	98,00	5000,00	135,00	2620,00	83,00
Patients	Hurley III	Female	28,00					
Patients	Hurley II	Male	45,00					
Patients	Hurley II	Female	44,00	63,00	1483,00	105,00	728,00	50,00
Patients	Hurley I	Male	29,00					
Patients	Hurley II	Female	40,00					
Patients	Hurley III	Male	46,00					

Patients	Hurley III	Female	41,00

il1lps1ada	il1candidaa	il1staphada	tnfmedium	tnflps1	tnfcandida	tnfstaph	tnfmedium	tnflps1ada
1293,00	60,00	305,00	153,00	1425,00	213,00	670,00	60,00	938,00
4780,00	65,00	1620,00	248,00	3593,00	278,00	1988,00	125,00	2618,00
5000,00	50,00	1780,00	315,00	350,00	275,00	945,00	85,00	350,00
4840,00	55,00	863,00	20,00	315,00				
1170,00	35,00	478,00	20,00	145,00				
2000,00	70,00	938,00	20,00	320,00				
20000,00	123,00	1128,00	20,00	725,00				
20000,00	50,00	1580,00	225,00					
			165,00	1170,00		340,00		
			20,00	2160,00		1260,00		
			20,00	1580,00		1510,00		
			20,00	175,00				
			20,00	1585,00		810,00		
			20,00	4800,00		580,00		
			1335,00			965,00		
			20,00	20,00		20,00	20,00	20,00
			20,00	1488,00				
120,00	20,00	150,00	80,00	80,00	80,00	180,00	80,00	80,00
			195,00	1260,00		580,00	1445,00	1100,00
			32,00	9530,00		5761,00	653,00	8503,00
			20,00	1429,00		20,00	20,00	143,00
			20,00	5080,00		1340,00	20,00	2720,00

			346,00	1450,00		20000,00	134,00	2037,00
			25,00	20,00		20,00	20,00	20,00
			20,00	20,00				
			2345,00	9840,00		10000,00	20,00	7180,00
			20,00	2165,00		1000,00		
			140,00	373,00		425,00		
5000,00	123,00	2980,00	20,00	20,00		20,00	95,00	20,00
735,00	120,00	2820,00						
1798,00	75,00	1961,00						
			544,00	1310,00		1786,00	868,00	622,00
			20,00	100,00		20,00	20,00	20,00
			20,00	6666,00		9217,00	1252,00	4998,00

			120,00	75,00				
			290,00	275,00	20,00	800,00	80,00	1380,00
			20,00	280,00		143,00	20,00	327,00
1298,00	50,00	523,00	80,00	438,00	95,00	545,00	80,00	355,00
12600,00	328,00	1980,00						
			20,00	8808,00		10000,00	395,00	3000,00
555,00	593,00	908,00	20,00	2362,00		20,00	20,00	1480,00
			20,00	327,00		349,00	20,00	20,00
			20,00	700,00		1340,00	20,00	340,00
2800,00	255,00	4300,00						
			246,00	984,00		959,00	228,00	402,00
			80,00	2948,00				
328,00	20,00	180,00	80,00	80,00	80,00	265,00	80,00	80,00

20,00	20,00	205,00	80,00	80,00	80,00	80,00	80,00	80,00
			20,00	2656,00				
			20,00	80,00		20,00	20,00	20,00
			20,00	1810,00		1150,00		
78,00	40,00	250,00	80,00	780,00	80,00	135,00	80,00	80,00
913,00	20,00	498,00	80,00	90,00	80,00	225,00	80,00	80,00
100,00	20,00	20,00	80,00	80,00	80,00	80,00	80,00	80,00
1475,00	178,00	2560,00	20,00	7800,00		10000,00	20,00	5180,00
			78,00	4193,00		7804,00	20,00	20000,00
			1420,00	1140,00		1460,00	20,00	20,00
			20,00	1475,00		5000,00		
			20,00	470,00		715,00		

			82,00	431,00		611,00	53,00	132,00
			20,00	928,00		723,00	20,00	362,00
			235,00	280,00		215,00		
			390,00	2573,00		3980,00		
85,00	20,00	95,00	80,00	115,00	80,00	2355,00	80,00	605,00
1960,00	150,00	125,00	80,00	1878,00	80,00	620,00	80,00	755,00
			20,00	1225,00		530,00		
			20,00	203,00		1320,00	20,00	100,00
			20,00	20,00		480,00	20,00	920,00
			20,00	540,00		20,00	180,00	320,00
2700,00	80,00	813,00						
1293,00	128,00	513,00						
2860,00	30,00	1103,00	718,00	2190,00	850,00	3728,00	605,00	2728,00
			140,00	1850,00		2260,00	35,00	10000,00
5000,00	115,00	2280,00	80,00	80,00		9733,00	20,00	1563,00
			20,00	120,00		20,00	20,00	20,00
1900,00	90,00	788,00						

tnfcandida	tnfstaphad	il6medium	il6lps1	il6candida	il6staph	il6medium	il6lps1ada	il22mediun
80,00	345,00	195,00	15500,00	10400,00	50,00	65,00	1500,00	115,00
263,00	1213,00	80,00		5200,00	7900,00	80,00	5700,00	410,00
80,00	895,00	90,00	20700,00	10200,00	25500,00	90,00	1735,00	2485,00
		20,00	5660,00	15450,00	19300,00	160,00		125,00
		20,00	25050,00	26400,00	23000,00	20,00		510,00
		20,00	73000,00	141000,00	143000,00	20,00		
		20,00		45000,00	139000,00	20,00		
		20,00		7410,00	109000,00			
	20,00							
		1090,00	8925,00					
80,00	155,00	90,00	1970,00	90,00	1730,00	90,00	820,00	1500,00
	140,00							380,00
								115,00
	7680,00							40,00
	20,00							
	860,00							

1281,00			
20,00			
			95,00
	20,00	3525,00	
10000,00			
			1325,00
			40,00
20,00			1490,00
1407,00			
20,00			
7442,00			

		20,00	180,00					
	480,00							
	120,00	20,00	5025,00					
80,00	395,00	625,00	3350,00	220,00	4535,00	160,00	4715,00	
	10000,00							1340,00
	20,00							
	20,00							215,00
	1040,00							
	857,00							
								240,00
		20,00	3945,00					
80,00	595,00	90,00	890,00	90,00	825,00	90,00	990,00	

80,00	80,00	90,00	90,00	90,00	230,00	90,00	90,00	40,00
								85,00
		20,00	5925,00					
80,00	80,00	90,00	500,00	90,00	270,00	90,00	130,00	
80,00	383,00	160,00	3250,00	90,00	2530,00	90,00	2230,00	
80,00	80,00	90,00	90,00	90,00	90,00	90,00	90,00	
	160,00							
	8515,00							40,00
								295,00
								135,00
	20,00							
								330,00
								290,00

	431,00							
	385,00							
80,00	1160,00	90,00	90,00	90,00	270,00	90,00	90,00	
								215,00
80,00	80,00	90,00	1515,00	90,00	230,00	90,00	1280,00	
								40,00
	200,00							
	240,00							
	20,00							
480,00	3813,00	90,00	12600,00	260,00	3670,00	90,00	6000,00	80,00
	1220,00							
	6551,00							145,00
	20,00							

il22candida	il22staph	il22mediun	il22candida	il22staphac	hbd2mediu	hbd2candic	hbd2staph	hbd2ada
115,00	315,00	40,00	135,00	220,00	5,00	5,00	5,00	5,00
2040,00	1485,00	145,00		1795,00	5,00	5,00	5,00	5,00
7295,00	3915,00	525,00	10000,00	3310,00	5,00	5,00	5,00	5,00
525,00	2160,00	40,00	375,00	2630,00	5,00	5,00	5,00	5,00
640,00	1015,00	40,00	590,00	950,00	5,00	5,00	5,00	5,00
795,00	385,00	100,00	915,00	1545,00	5,00	5,00	5,00	5,00
2945,00	1370,00	475,00	4895,00	2440,00	5,00	5,00	5,00	5,00
280,00	125,00	40,00	40,00	40,00	5,00	5,00	5,00	5,00
340,00	1135,00	40,00	465,00	855,00	5,00	5,00	5,00	5,00

1005,00	85,00	40,00	400,00	105,00	5,00	5,00	5,00	5,00
1690,00	1250,00	365,00	1000,00	1490,00	5,00	5,00	5,00	5,00
110,00	40,00	40,00	125,00	565,00	5,00	5,00	5,00	5,00
10000,00	10000,00	2635,00	7970,00	9665,00	5,00	5,00	5,00	5,00

3605,00	955,00	1000,00	3200,00	795,00	5,00	5,00	5,00	5,00
895,00	580,00	150,00	1045,00	980,00	5,00	5,00	5,00	5,00
1515,00	710,00	40,00	2495,00	510,00	5,00	5,00	5,00	5,00

370,00	40,00	40,00	495,00	40,00	5,00	5,00	5,00	5,00
1115,00	1585,00	115,00	9045,00	1210,00	5,00	5,00	5,00	5,00
150,00	630,00	40,00	40,00	415,00	5,00	5,00	5,00	5,00
1320,00	2430,00	265,00	1625,00	2165,00	5,00	5,00	5,00	5,00
290,00	610,00	125,00	345,00	615,00	5,00	5,00	5,00	5,00
740,00	2330,00	225,00	1625,00	3720,00	5,00	5,00	5,00	5,00
295,00	370,00	240,00	895,00	520,00	5,00	5,00	5,00	5,00

140,00	215,00	170,00	215,00	270,00	5,00	5,00	5,00	5,00
345,00	675,00	105,00	165,00	925,00	5,00	5,00	5,00	5,00
180,00	145,00	40,00	450,00	180,00	5,00	5,00	5,00	5,00
5470,00	4940,00	190,00	3205,00	4220,00	5,00	5,00	5,00	5,00

hbd2candid	hbd2staph	il17mediun	il17lps	il17candida	il17staph	il17rpmihu	il17lpshum	il17candida
5,00	5,00	78,00	78,00	78,00	78,00			
	5,00	78,00	78,00	65,00	99,00			
5,00	5,00	78,00	78,00	78,00	43,00			
5,00	5,00	78,00	190,00	291,00	920,00	1230,00	420,00	5180,00
100,00	5,00	78,00	220,00	2610,00	1380,00	78,00	380,00	364,00
		78,00	78,00	900,00	1740,00	89,00	180,00	1520,00
		350,00	150,00	320,00	640,00	230,00	105,00	320,00
		78,00	78,00	78,00	78,00	78,00	78,00	78,00
		120,00	50,00	595,00	165,00	110,00	78,00	535,00
		78,00	78,00	55,00	78,00	78,00	78,00	60,00
		1535,00	220,00	4890,00	4750,00	1430,00	175,00	3440,00
		78,00	78,00	95,00	130,00	78,00	78,00	85,00
		230,00	235,00	870,00	2640,00	210,00	165,00	845,00
		78,00	78,00	78,00	130,00	78,00	78,00	78,00
		305,00	135,00	1350,00	3270,00	155,00	55,00	800,00
		78,00	78,00	45,00	90,00	78,00	41,00	78,00
		78,00	90,00	45,00	300,00			
5,00	5,00	50,00	270,00	580,00	765,00	70,00	215,00	890,00
5,00	5,00	78,00	78,00	410,00	78,00			
		43,00	78,00	78,00	180,00			
5,00	5,00	78,00	78,00	70,00	65,00	78,00	195,00	100,00
5,00	5,00	540,00	675,00	635,00	3770,00	980,00	700,00	930,00

5,00	5,00	78,00	78,00	400,00	78,00	78,00	78,00	580,00
		78,00	78,00	240,00	800,00	130,00	190,00	540,00
		78,00	310,00	300,00	590,00	280,00	79,00	330,00
		78,00	78,00	370,00	460,00			
5,00	5,00	250,00	55,00	545,00	260,00	280,00	135,00	470,00
5,00	5,00							
		78,00	78,00	78,00	75,00	190,00	75,00	325,00
		78,00	78,00	78,00	110,00			
5,00	5,00	78,00	78,00	78,00	78,00			
		78,00	78,00	78,00	78,00	78,00	78,00	78,00

		78,00	78,00	180,00	130,00	78,00	78,00	460,00
		78,00	78,00	78,00	78,00			
		50,00	78,00	290,00	260,00	115,00	55,00	495,00
		410,00	78,00	255,00	100,00	260,00	78,00	185,00
5,00	5,00	81,00	170,00	420,00	760,00			
5,00	5,00	200,00	75,00	290,00	270,00	180,00	170,00	555,00
		1735,00	955,00	1890,00	760,00	1970,00	570,00	1600,00
5,00	5,00	235,00	100,00	480,00	820,00	180,00	78,00	3205,00
		78,00	83,00	135,00	78,00	78,00	78,00	78,00
		78,00	78,00	78,00	78,00			

		140,00	78,00	380,00	600,00	240,00	310,00	480,00
		80,00	78,00	135,00	80,00	78,00	78,00	95,00
		42,00	78,00	78,00	78,00	78,00	78,00	80,00
5,00	5,00							
5,00	5,00	87,00	78,00	330,00	450,00	330,00	78,00	490,00
		78,00	50,00	935,00	1050,00	78,00	78,00	1155,00
		50,00	120,00	1695,00	1690,00	78,00	295,00	1680,00
		720,00	270,00	1450,00	810,00	550,00	350,00	1140,00
5,00	5,00							
		110,00	78,00	1220,00	3380,00	270,00	320,00	1680,00
5,00	5,00	220,00	90,00	290,00	800,00	200,00	78,00	515,00
5,00	5,00	78,00	78,00	78,00	78,00	78,00	78,00	400,00
		454,00	85,30	25,10	436,00	16,60	8,00	0,81
		298,00	0,28	25,80	293,00	19,50	2,00	1,23
5,00	5,00	78,00	78,00	80,00	180,00	78,00	44,00	550,00
		86,00	130,00	510,00	3260,00	730,00	210,00	1950,00
5,00	5,00							

		170,00	775,00	2355,00	360,00	860,00	615,00	1815,00
		610,00	190,00	1395,00	1595,00	525,00	16,00	1520,00
5,00	5,00	78,00	78,00	78,00	78,00	78,00	78,00	78,00
5,00	5,00	47,00	44,00	230,00	265,00	110,00	140,00	405,00
		78,00	78,00	97,00	200,00	200,00	78,00	350,00
		550,00	78,00	930,00	295,00	665,00	150,00	840,00
		180,00	250,00	1510,00	1850,00			
		78,00	78,00	78,00	78,00	78,00	78,00	110,00
5,00	5,00	65,00	80,00	315,00	215,00	100,00	78,00	280,00
5,00	5,00	100,00	140,00	350,00	660,00	78,00	230,00	660,00

il17staphhu	il1ramadiu	il1ralps	il1rastaph	il1raada	il1ralpsada	il1rastapha	il10mediun	il10lps
	4445,00		8695,00	4595,00		8090,00	20,00	6820,00
	7195,00		8665,00	6765,00		7655,00	20,00	5780,00
	5650,00		8250,00	6275,00		8000,00	20,00	5500,00
2150,00	6110,00		8785,00	5340,00		8610,00	20,00	6720,00
1720,00	4685,00		8665,00	4745,00		7420,00		5740,00
2550,00	8786,00		8030,00	4375,00		7210,00		6970,00
545,00	52565,00		54408,00					6580,00
78,00	64179,00		65285,00					6130,00
130,00	20028,00		56068,00					10,00
78,00	51275,00		66852,00					60,00
3160,00	9889,00		68972,00					
135,00	41043,00		28324,00					
2390,00	46113,00		57727,00					
80,00	9613,00		56897,00					
2720,00	1090,00		42426,00					
41,00	1090,00		64916,00					
	6144,00	3616,00	14000,00	6520,00	7246,00	7524,00	10,00	1470,00
1595,00								
							200,00	3000,00
120,00								
3350,00	14000,00	14000,00	6028,00	218,00	14000,00	14000,00	10,00	40,00
	6376,00	7516,00	218,00	218,00	6318,00	3568,00	10,00	750,00
							25,00	25,00

	235,00	3000,00
160,00		
3430,00		
970,00		
335,00		
455,00		
78,00		
	10,00	890,00
	10,00	10,00

270,00								
	6634,00	6346,00	7990,00	6086,00	7802,00	6818,00	10,00	495,00
	3914,00	7672,00	4952,00	3060,00	218,00	218,00	10,00	165,00
475,00								
355,00								
	218,00	218,00	17380,00	14000,00	14000,00	14000,00	10,00	85,00
							10,00	465,00
395,00	6770,00	7940,00	8030,00	6260,00	218,00	218,00	210,00	205,00
	7410,00	14000,00	14810,00	6472,00	218,00	218,00	245,00	190,00
640,00								
							165,00	500,00
1095,00								
275,00								
	14000,00	8866,00	218,00	218,00	14000,00	14000,00	10,00	490,00

830,00								
105,00								
65,00								
460,00								
1575,00								
1095,00								
740,00								
	4298,00	218,00	14000,00	7778,00	7434,00	7082,00	10,00	10,00
							10,00	220,00
4180,00								
720,00								
260,00								
4,40								
3,57								
1060,00								
2750,00								

	1786,00	218,00	14000,00	4914,00	6740,00	6510,00	10,00	190,00
							10,00	105,00
460,00								
1570,00								
78,00								
410,00								
							10,00	255,00
							10,00	10,00
	2304,00	2074,00	14000,00	14000,00	14000,00	14000,00	100,00	3000,00
260,00								
175,00								
84,00								
230,00			7110,00				10,00	80,00
			13740,00				10,00	230,00
1780,00			16920,00				230,00	365,00
	7818,00	6626,00	14960,00	14000,00	14000,00	14000,00	10,00	30,00

il10staph	il10candida	il10medada	il10lpsada	il10staphac	il10candida	il1bgene	tnfgene	il1rapus
7500,00	7380,00					2,40	13,54	
7040,00	7390,00					14,51	1,79	
6490,00	6090,00					0,77	1,93	
7000,00	7330,00							
5930,00	6050,00							
6370,00	6820,00							
5720,00	7120,00							
6250,00	4890,00							
10,00	10,00							
520,00	1180,00							
210,00		10,00	850,00	10,00				
						0,21	0,04	
	10,00				10,00			
250,00		10,00	555,00	10,00				51551,00
								69709,00
								80239,00
10,00		10,00	160,00	60,00				
285,00		10,00	1075,00	230,00				
10,00		10,00	10,00	10,00				

250,00		80,00	3000,00	200,00				
						14,62	8,25	16710,00
								30293,00
								59939,00
	10,00				10,00			77778,00
	10,00				10,00			
	10,00				10,00			
								77472,00
								72887,00
760,00		40,00	425,00	255,00				
70,00		10,00	30,00	10,00				

						0,00	0,00	218,00
10,00		10,00	235,00	10,00				
10,00		10,00	215,00	430,00		2,07	2,62	90279,00
	10,00				10,00			
	10,00				10,00			
330,00		10,00	15,00	10,00				
135,00		10,00	435,00	80,00				
50,00		10,00	10,00	10,00				36619,00
480,00		10,00	295,00	435,00				
	10,00				10,00			
390,00		10,00	3000,00	3000,00				
								6078,00
						5,88	6,96	
65,00		10,00	545,00	135,00		0,51	6,86	88302,00

	10,00				10,00			
								87986,00
						38,68	5,76	69064,00
								85061,00
								13950,00
	10,00				10,00			59974,00
								1133,00
	10,00				10,00			
	10,00				10,00			
10,00		10,00	10,00	10,00				
225,00		10,00	255,00	10,00				
								98658,00
								61782,00

10,00		10,00	100,00	10,00		
95,00		190,00	20,00	10,00		
						8691,00
						92650,00
	10,00				10,00	
	10,00				10,00	
260,00		10,00	775,00	495,00		
370,00		3000,00	10,00	10,00		
1160,00		10,00	580,00	65,00		
	10,00				10,00	
	10,00				10,00	
						85773,00
10,00		10,00	55,00	55,00		68419,00
160,00		10,00	260,00	10,00		
150,00		10,00	265,00	180,00		39200,00
545,00		10,00	315,00	115,00		
						87986,00

il10pus	tnfpus	il1bpus	il6pus	il1alphapus	il17pus	monocytes	countcd14	countcd14l
						368,00	338,00	9,00
						438,00	412,00	4,00
						637,00	600,00	7,00
						786,00	728,00	7,00
						600,00	580,00	1,00
						337,00	312,00	3,00
						676,00	645,00	5,00
						351,00	323,00	5,00
						284,00	244,00	5,00
						507,00	481,00	3,00
						91,00	80,00	1,00
						353,00	307,00	9,00
						186,00	169,00	3,00
						181,00	150,00	6,00
						576,00	434,00	21,00
						2097,00	1937,00	41,00
						2018,00	1858,00	20,00
						564,00	533,00	2,00
						545,00	504,00	26,00
420,00	45500,00	10100,00	910,00	220,00	2960,00	764,00	670,00	8,00
10,00	20,00	16900,00	20,00					
						429,00	374,00	23,00
15,00	400,00	7890,00	910,00	140,00	80,00			
						450,00	413,00	6,00
						384,00	314,00	6,00
						1027,00	946,00	11,00
						647,00	531,00	11,00

						870,00	839,00	2,00
						624,00	586,00	4,00
10,00	2200,00	80,00	590,00	40,00	80,00	402,00	375,00	3,00
						351,00	335,00	4,00
						424,00	385,00	6,00
10,00	2700,00	30300,00	1510,00	1060,00	4220,00	558,00	507,00	13,00
						469,00	412,00	15,00
10,00	3600,00	27900,00	1500,00	760,00	80,00	396,00	358,00	9,00
						345,00	314,00	4,00
495,00	10000,00	83500,00	930,00	440,00	240,00	1426,00	1343,00	19,00
						335,00	278,00	4,00
10,00	400,00	8000,00	500,00	320,00	80,00	1393,00	28,00	43,00
						697,00	665,00	6,00
						691,00	611,00	19,00
10,00	20,00	3500,00	20,00					
						634,00	537,00	12,00
						1080,00	977,00	17,00

95,00	400,00	4600,00	310,00	260,00	80,00			
						600,00	554,00	13,00
10,00	400,00	4050,00	2470,00	80,00	80,00			
						689,00	45,00	56,00
10,00	20,00	2020,00	740,00	40,00	80,00	854,00	815,00	3,00
						504,00	467,00	5,00
						544,00	474,00	11,00
						630,00	583,00	12,00
						302,00	276,00	4,00
						595,00	554,00	7,00
10,00	800,00	2060,00	350,00	40,00	80,00			
						482,00	462,00	2,00
210,00	33700,00	47300,00	650,00	320,00	80,00	1179,00	17,00	53,00

						1422,00	1331,00	18,00
10,00	20,00	9760,00	200,00	100,00	80,00			
10,00	2400,00	44500,00	150,00	120,00	860,00	795,00	748,00	7,00
10,00	400,00	22500,00	1000,00	360,00	80,00			
40,00	13200,00	4000,00	20,00			904,00	858,00	12,00
						718,00	656,00	13,00
						412,00	339,00	9,00
						463,00	408,00	6,00
						372,00	340,00	5,00
10,00	245,00	7700,00	200,00	100,00	40,00	783,00	726,00	14,00
10,00	2500,00	19900,00	20,00					
						370,00	340,00	5,00
						316,00	305,00	2,00
						625,00	563,00	18,00
90,00	80,00	54000,00	1800,00					
						318,00	268,00	7,00
						180,00	172,00	2,00
10,00	900,00	19600,00	400,00					

						508,00	457,00	9,00
						467,00	423,00	9,00
90,00	2500,00							
10,00	40700,00	24200,00	500,00					
						527,00	497,00	3,00
						425,00	370,00	13,00
						324,00	303,00	3,00
						449,00	395,00	20,00
						634,00	600,00	4,00
						479,00	408,00	8,00
						493,00	441,00	13,00
						405,00	365,00	3,00
10,00		15000,00						
10,00	18600,00	8200,00	250,00	140,00	80,00	1053,00	855,00	55,00
						451,00	410,00	7,00
10,00	7100,00	6220,00	700,00	40,00	80,00	405,00	372,00	4,00
10,00	1500,00	49900,00	700,00					

978,00	916,00	6,00

countcd14l	countcd14(	countcd14negcd16bright
13,00	5,00	4,00
17,00	5,00	0,00
18,00	3,00	2,00
33,00	13,00	9,00
13,00	2,00	1,00
13,00	5,00	2,00
15,00	4,00	2,00
15,00	3,00	3,00
26,00	4,00	10,00
15,00	3,00	1,00
3,00	3,00	3,00
18,00	15,00	9,00
6,00	5,00	2,00
7,00	8,00	5,00
83,00	26,00	6,00
75,00	21,00	5,00
80,00	33,00	9,00
16,00	6,00	4,00
7,00	3,00	1,00
42,00	17,00	16,00
25,00	5,00	2,00
21,00	5,00	2,00
33,00	23,00	21,00
40,00	6,00	4,00
41,00	69,00	21,00

7,00	3,00	1,00
14,00	3,00	1,00
11,00	5,00	1,00
5,00	2,00	1,00
14,00	9,00	3,00
23,00	6,00	6,00
25,00	8,00	9,00
15,00	8,00	5,00
9,00	12,00	5,00
28,00	12,00	7,00
35,00	11,00	11,00
9,00	2,00	
9,00	3,00	1,00
39,00	12,00	7,00
61,00	20,00	11,00
61,00	11,00	8,00

15,00	3,00	2,00
29,00	25,00	20,90
14,00	4,00	3,00
12,00	5,00	1,00
35,00	17,00	9,00
12,00	11,00	2,00
15,00	4,00	4,00
19,00	6,00	2,00
10,00	3,00	5,00
26,00	7,00	

53,00	6,00	4,00
19,00	11,00	6,00
15,00	3,00	2,00
26,00	10,00	2,00
24,00	37,00	42,00
21,00	21,00	12,00
14,00	8,00	6,00
23,00	10,00	2,00
10,00	8,00	11,00
5,00	3,00	1,00
35,00	8,00	6,00
24,00	13,00	2,00
2,00	1,00	1,00

27,00	5,00	0,00
18,00	8,00	6,00
10,00	4,00	2,00
23,00	11,00	5,00
7,00	3,00	1,00
24,00	3,00	2,00
15,00	8,00	2,00
33,00	19,00	5,00
23,00	9,00	6,00
24,00	9,00	3,00
97,00	21,00	6,00
17,00	7,00	4,00
12,00	6,00	4,00

14,00	24,00	8,00
